# Multizonal anogenital neoplasia in women: a cohort analysis

**DOI:** 10.1186/s12885-021-07949-8

**Published:** 2021-03-06

**Authors:** Andreia Albuquerque, Michelle A. L. Godfrey, Carmelina Cappello, Francesca Pesola, Julie Bowring, Tamzin Cuming, Anke De Masi, Adam N. Rosenthal, Peter Sasieni, Mayura Nathan

**Affiliations:** 1 Homerton Anogenital Neoplasia Service (HANS), Homerton University Hospital, London, UK; 2 Department of Obstetrics and Gynaecology, Queen Alexandra Hospital, Portsmouth, UK; 3 King’s College London, London, UK; 4 University College Hospital NHS Foundation Trust, London, UK

**Keywords:** anogenital neoplasia, women, multizonal anogenital neoplasia, high-grade squamous intraepithelial lesions, anogenital cancer

## Abstract

**Background:**

There is currently a lack of information on full anogenital evaluation of women with a previous history of anogenital neoplasia.

**Methods:**

Retrospective analysis of the Homerton Anogenital Neoplasia Service records from January 2012 to March 2017, to identify all new referrals of women with previous anogenital neoplasia, who had had at least one complete examination of all anogenital sites. Multizonal anogenital disease (MZD) was defined as the presence of high-grade squamous intraepithelial lesions (HSIL)/carcinoma concurrently at two or more of the following sites/zones: perianus, anal canal, vulva, vagina or cervix.

**Results:**

253 women were included, mean age was 47 (SD=15) years and median duration of follow-up was 12 (IQR=21) months. Fifty-six women (22%) were diagnosed with MZD at first assessment and/or during follow-up. Current smokers (RR=1.84, 95% CI 1.21–2.79, p=0.004) and women on immunodulators/immunosuppressive drugs (RR=2.57, 95% CI 1.72-3.86, p<0.001) had an increased risk for MZD. The risk was lower for women without a previous history of anogenital high-grade lesions/cancer compared to those with this history (RR=0.06, 95% CI 0.01-0.45, *p*=0.006).

**Conclusions:**

Multizonal assessment was important to diagnose occult areas of disease and should be especially considered in current smokers, pharmacologically immunocompromised and those with a previous history of anogenital HSIL/cancer.

**Supplementary Information:**

The online version contains supplementary material available at 10.1186/s12885-021-07949-8.

## Introduction

Women with high-risk human papillomavirus infections are at risk of high-grade intraepithelial neoplasia (HSIL) and squamous cell carcinoma (SCC) of the lower anogenital tract [1]. Some women present with disease at only one anogenital site, but go on to develop disease in several anogenital zones [2].

Women with a history of genital neoplasia or cancer have a higher risk of anal neoplasia [3–10], with the link between cervical and anal cancer well described. The risk of anal neoplasia increases with the number of anogenital sites involved, and there is evidence of preferential site involvement; the risk of anal canal neoplasia is greatest in patients with perianal disease, but is also elevated in those with vulval, vaginal and cervical neoplasia [4, 10].

The Lower Anogenital Squamous Terminology (LAST) recommends that squamous intraepithelial lesions are classified as low-grade (LSIL) and high-grade (HSIL) for all anogenital sites [11]. HSILs have a higher risk of cancer progression compared with LSILs; therefore, different therapeutic and follow-up approaches are recommended for these lesions [12]. Despite several studies evaluating the prevalence and risk factors for anal HSIL and SCC in women with a previous history of genital neoplasia, information on women undergoing a full anogenital assessment (defined as cervix, vagina, vulva, perianus and anal canal) at the same examination is scarce. Additionally, information relating to perianal disease as a site of involvement is rare, partly due to inclusion of this area either with genital sites or with the anal canal in published research [4]. Long-term follow-up information is also lacking. Multizonal anogenital neoplasia (MZN) assessment (defined as full anogenital evaluation at the same examination to include the cervix, vagina, vulva, perianus and the anal canal) might be essential to diagnose occult areas of HSIL or carcinoma, given the risk that such lesions are likely to be present at more than a single anogenital site. A recent meta-analysis found an increased incidence of second HPV-driven cancers in a population with previous HPV-associated cancers when compared to controls [13].

In women with anogenital neoplasia, identification of risk factors for the involvement of more than one site is important to determine which patients might benefit the most from a full multizonal anogenital assessment. Here we present the findings in a cohort of women referred with a history of anogenital neoplasia including LSIL, HSIL and/or carcinoma, and examined with multizonal high-resolution assessment at a single tertiary reference centre.

## Methods

### Study design, inclusion and exclusion criteria

The study consisted of a retrospective analysis of the medical records from the Homerton Anogenital Neoplasia Service in London, between January 2012 to March 2017, to identify all new referrals of women with a history of anogenital neoplasia who underwent at least one MZN assessment. The study inclusion criteria were 1) new referral in the period from January 2012 to March 2017; 2) a previous history of anogenital neoplasia (LSIL including warts, HSIL and/or cancer) and 3) at least one MZN assessment (during their visits). Women referred and evaluated outside this period, and those with incomplete information on the medical records regarding MZN assessment were excluded. The Health Research Authority approved this study (IRAS number 229262) after the London - Surrey Borders Research Ethics Committee approval (17/LO/1159). All methods were performed in accordance with the relevant guidelines and regulations. Informed consent was waived off, this was a retrospective study of existing data. Any patient that has explicitly expressed dissent in his/her clinical consent form in their medical records that did not wish for their records to be used for research purposes were not included in this study. The anonymized dataset used and analysed during the current study is available from the corresponding author on reasonable request.

### Definition and outcomes

Multizonal anogenital neoplasia (MZN) was defined as the presence of HSIL or SCC during the same examination in at least two of the following anatomical zones: perianus, anal canal, vulva, vagina or cervix. The perianus was defined as an area 5cm around the anal margin, except anteriorly, where the limit was the posterior border of the fourchette or 5cm, whichever was closer.

The primary outcome was to evaluate the risk factors for anogenital MZN in a cohort of women with a previous history of anogenital neoplasia.

### Multizonal anogenital neoplasia assessment

Multizonal anogenital neoplasia assessment consists of examination using magnification with an Olympus® colposcope (Tokyo, Japan) of the anogenital sites after the application of 5% acetic acid. Patients are examined in the lithotomy position with the examination commencing with the cervix using a bivalve speculum. Examination is continued to include all vaginal surfaces from the fornices to the introitus. Biopsies were obtained from areas suspicious of HSIL. Further examination includes the introitus and the vulval region. Examination of the perianus follows and all areas of suspected HSIL are recorded on a diagram for future reference. The last phase of the examination includes the anal canal, using an anoscope with 5% acetic acid application (high-resolution anoscopy).

Biopsies were obtained using Tischler punch-biopsy forceps in areas suspicious for HSIL and/or cancer after local anaesthesia. Haemostasis was achieved with the application of Monsel’s solution (ferric subsulphate) via a cotton-bud when needed. Clinicians who conducted the examinations had all been trained under direct supervision by M.N. and were experienced in MZN assessment. Women with a high burden of disease (large surface area involved), significant discomfort or with suspected cancer, were offered examination under general anaesthesia. In all cases, the lesion distribution for each zone was recorded, along with the precise location of biopsies taken. Anal cytology was routinely collected for all patients, but this data was not included in this study.

### Histology

For the histological classification of the lesions, the terms LSIL and HSIL were used [11]. p16 immunohistochemical stain positive -IN2 lesions were considered HSIL and -IN2 p16-negative lesions as LSIL. Condylomas were considered LSIL [11]. In historical cases in which p16 immunohistochemistry was not performed, -IN2 lesions were considered HSIL.

### Statistical analysis

Categorical variables were described as absolute numbers, and relative frequencies and continuous variables were described as mean (standard deviation, SD) or median (interquartile range, IQR), according to the distribution symmetry. Comparisons between patients with and without MZN were performed using the Mann-Whitney U test for continuous variables and the chi-square test for categorical variables. For current smokers, the number of pack-years was calculated as the number of cigarettes smoked per day/20 × number of years smoked. Relative risk (RR) and corresponding 95% confidence intervals (CI) were estimated running a log-binomial regression using a generalized linear model in Stata version 13 (Stata Corporation, College Station, Texas, USA). Factors that were found to be statistically significant in the univariate model were simultaneously included in a multivariate regression model. A significance level of α=5% was considered in all hypothesis tests.

## Results

From January 2012 to March 2017, 308 new female referrals were seen at the study Centre. Two hundred and seventy-one women had information relating to MZN evaluation and, in 253 of these cases, MZN assessment was complete and was included in the final analysis, Figure S1 (Supporting Information). The median age at first visit was 47 (IQR=36-56) years, and the median duration of follow-up was 12 (IQR=21) months, Table 1. In total, 191 of the 253 women (75%) had a previous history of anogenital neoplasia (HSIL and/or cancer) at any site, including 51 cases (20%) of anogenital cancer. The most common previously affected site was the cervix in 94/251 (37%). Eighty-nine (37%) of 240 women (for whom this information was available) were current smokers, 33 (13%) were HIV-positive, 35 (14%) were pharmacologically immunocompromised (on immunomodulators/immunosuppressive drugs) and 37 of 128 (29%) women in whom the information was available reported having had anal sexual intercourse. The main reasons for referral were a history of anogenital neoplasia and/or a request for MZN evaluation (75%) or management of anogenital warts (22%).

**Table 1:** Characteristics of the women with multizonal anogenital neoplasia assessment included in this study (*n*=253)

Parameter	Descriptive statistics^a^
Age at first visit (years) median (IQR)	47 (36-56)
Duration of the follow-up (months) median (IQR)	12 (21)
Previous cervical HSIL/cancer, n (%)	94/251 (37)
Previous vulval HSIL/cancer, n (%)	91 (36)
Previous vaginal HSIL/cancer, n (%)	16 (6)
Previous anal HSIL/cancer, n (%)	62 (25)
Previous perianal HSIL/cancer, n (%)	34 (13)
Previous history of anogenital tract HSIL/cancer any site, n (%)	191(75)
One site HSIL/cancer, n (%)	112 (59)
Two sites HSIL/cancer, n (%)	61 (32)
Three sites HSIL/cancer, n (%)	14 (7)
Four sites HSIL/cancer, n (%)	3 (1.5)
Five sites HSIL/cancer, n (%)	1 (0.5)
Previous anogenital cancer/per patient, n (%)	51 (20)
Cervical	9
Vulval	16
Anal	21
Perianal	8
One anogenital cancer site, n (%)	48 (94)
Two anogenital cancers sites, n (%)	3 (6)
Ever smoking, n (%)	133/235 (57)
Current smoking, n (%)	89/240 (37)
HIV-positive, n (%)	33 (13)
Pharmacologically immunocompromised, n (%)	35 (14)
Anal sexual intercourse, n (%)	37/128 (29)
MZN at first visit and/or follow-up, n (%)	56 (22)
Referral Centre first visit (*n*=253)/ MZN assessment first visit (*n*=252)	
MZN diagnosis, n (%)	50/252 (20)
Any site with HSIL/cancer	107 (42)
HSIL/cancer diagnosis in a new site/per patient, n (%)	53 (21)
Anogenital cancers/per patient, n (%)	9 (4)
New cases (in other sites) anogenital cancers/per patient, n (%)	6 (2)
Patients with follow-up (*n*=184)/ MZN assessment follow-up (*n*=180)	
MZN diagnosis, (%)	20/180 (11)
New MZN diagnosis, n (%)	6/180 (3)
Any site with HSIL/cancer, n (%)	50 (27)
New HSIL/cancer in new areas/per patient, n (%)	32 (17)
Anogenital cancers/per patient, n (%)	4 (2)
New anogenital cancers (in other sites)/per patient, n (%)	4 (2)

*HSIL* High-grade squamous intraepithelial lesions, *IQR* Interquartile range, *MZN* Multizonal anogenital neoplasia, *SD* Standard deviation

^a^Median (IQR) reported for continuous variables while N (%) are reported for categorical variables. The % for categorical variables is calculated using 253 as the denominator unless otherwise specified in the cell. Denominator may vary due to missing data

Fifty-six women (22%) were diagnosed with MZN at first assessment or during the follow-up. Multizonal disease was diagnosed in 50 patients (20%) at the first visit, the most commonly affected sites were the perianus (40 cases) and anal canal (34 cases). Most patients with MZN had involvement of two sites (27 cases, 54%), Table 2. At first visit, HSIL /cancer at any site was diagnosed in 107/253 patients (42%) and in 53 (21% of all patients) this corresponded to a new or unsuspected zone of disease at referral. Anogenital cancer was diagnosed at the initial visit in 9 women (4%) and in 6 (2%) this was a cancer in a new and previously unsuspected zone, Table 1.

**Table 2:** Description of multizonal anogenital neoplasia cases at first visit and during the follow-up by site location and number of sites

**MZN At first visit (*n*=50)**	
**Disease location**	
**Site**	**Number of cases HSIL/cancer**
Cervical	3
Vulval	37
Vagina	14
Anal	34
Perianal	40
**Number of sites HSIL/cancer**	**N (%)**
Two sites	27 (54)
Three sites	18 (36)
Four sites	5 (10)
Five sites	0 (0)
**MZN at follow-up (*n*=20)**	
**Disease location**	
**Site**	**Number of cases HSIL/cancer**
Cervical	2
Vulval	12
Vagina	8
Anal	13
Perianal	13
**Number of sites HSIL/cancer**	**N (%)**
Two sites	13 (65)
Three sites	6 (30)
Four sites	1 (5)
Five sites	0 (0)

(Multizonal anogenital neoplasia involves, by definition, two or more anatomical zones).

*HSIL* High-grade squamous intraepithelial lesions, *MZN* Multizonal anogenital neoplasia

Follow-up information was recorded for 184/253 women (73%), 180 of whom had MZN assessment at first visit. Multizonal anogenital neoplasia was identified during follow-up in 20 patients (11%), and in 6 (3%) this was a new MZN diagnosis. The most commonly affected sites were perianal (13 cases) and anal (13 cases), Table 2. HSIL/cancer at any site was diagnosed during follow-up in 50 women (27%), and in 32 (17%) this corresponded to a new area of disease. Anogenital cancer was diagnosed as a new site of cancer in 4 women (2%) at follow-up, Table 1.

A comparison between patients with (*n*=56) and without MZN (*n*=197) was made (Table 3). Patients with MZN were more commonly on immunomodulators / immunosuppressive drugs (27% vs 10% *p*=0.001), current smokers (51% vs 33% *p*=0.021) and/or had a previous history of HSIL/cancer at any of the anogenital zones (98% vs 69% *p*<0.001). There was no significant difference between the two groups regarding age at first visit, age at first HSIL/cancer diagnosis, current smoking pack-years, HIV-positivity, anal sexual intercourse, time since the first anogenital HSIL/cancer to the first visit, and the first HSIL/cancer site.

**Table 3:** Risk factors associated with multizonal anogenital neoplasia diagnosis at first assessment and/or follow-up

	MZN (*n*=56)	NON-MZN (*n*= 197)	Test^a^	*p*-value
Age at first visit (years), median (IQR)	46 (39-55)	47 (35-57)	Z= -0.03^1^	0.98
Age at first HSIL/cancer (years), median (IQR)	41 (34-49)	43 (32-55)	Z=1.09^1^	0.278
Ever smoked, n (%)	31 (61)	102 (55)	Chi^2^(1)=0.47^2^	0.50
Current smoker, n (%)	26 (51)	63 (33)	Chi^2^(1)=5.4^2^	0.02
Current smoking pack-years, median (IQR)	15 (11-21)	22 (9-35)	Z=0.531	0.60
HIV-positive, n (%)	11 (20)	22 (11)	Chi^2^(1)=2.8^2^	0.10
Pharmacologically immunocompromised, n (%)	15 (27)	20 (10)	Chi^2^(1)=10.1^2^	0.001
Anal sexual intercourse, n (%)	5 (21)	32 (31)	Chi^2^(1)=0.9^2^	0.33
Time since the 1^st^ anogenital HSIL/cancer diagnosis (years) to first visit, median (IQR)	3 (1-9)	4 (1-12)	Z=1.31	0.19
Previous history of anogenital cancer, n (%)	14 (25)	37 (19)	Chi^2^(1) = 1.1^2^	0.31
Previous history of HSIL/cancer any site, n (%)	55 (98)	136 (69)	Chi^2^(1)=20.1^2^	<0.001
Previous history of HSIL/cancer by first site				
Cervical, n (%)	28 (51)	64 (47)	Chi^2^(4)=7.7^2^	0.10
Vulval, n (%)	19 (35)	30 (22)		
Vaginal, n (%)	0 (0)	5 (4)		
Anal, n (%)	7 (13)	27 (20)		
Perianal, n (%)	1 (2)	10 (7)		

*HSIL* High-grade squamous intraepithelial lesions, *IQR* Interquartile range, *MZN* Multizonal anogenital neoplasia cases, *NON-MZN* Cases with no multizonal anogenital neoplasia ever diagnosed

^a^ We used 1) Mann-Whitney test for continuous outcomes; or 2) Chi-square test for binary outcome.

**Fig. 1:**
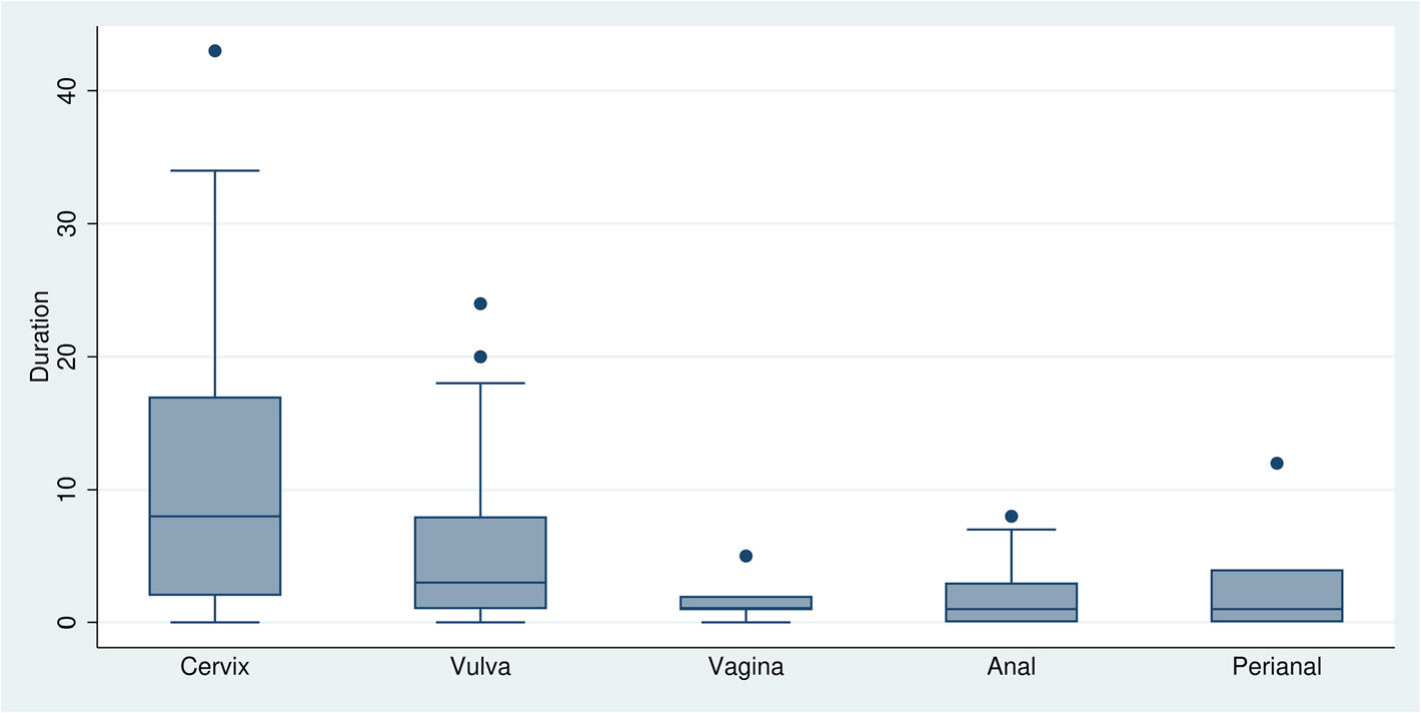
Time since the first diagnosis of high-grade squamous intraepithelial lesions/cancer (in years) to the first visit at the referral centre, by anogenital site

Women with a previous HSIL/cancer history, in whom the cervix had been the first affected zone (index zone), were referred after a longer interval than women who had had a first HSIL/cancer at any other zone (Fig. 1, *p*=0.001). For a cervical index lesion, the median time (in years) for referral was 8(IQR=15), for the vulva it was 3 (IQR=7), for the vagina 1 (IQR=1), for the anal canal 1 (IQR=3) and for the perianus it was 1 (IQR=4).

The multivariate regression model showed an increased risk for MZN in current smokers (RR=1.84, 95% CI 1.21–2.79, *p*=0.004) and in women on immunomodulators/immunosuppressive drugs (RR=2.57, 95% CI 1.72-3.86, *p*<0.001). The risk was lower for women without a previous history of HSIL/cancer compared to those with this history (RR=0.06, 95% CI 0.01-0.45, *p*=0.006). Lastly, we conducted an exploratory analysis where we regressed MZN on previous history of HSIL/cancer and a composite score which coded whether women met one of the following criteria, women who were HIV-positive, pharmacologically immunosuppressed or current smokers. Results from this model showed that the risk for MZN was higher (RR=2.53, 95% CI 1.45 to 4.40, *p*=0.001) in women who met one of these criteria compared to those who did not have any of these risk factors. A lower risk was noted for women without a previous history of HSIL/cancer (RR = 0.06, 95% CI: 0.01 to 0.40, *p* =0.004).

## Discussion

Studies of women with complete anogenital evaluation for neoplasia during a single examination are scarce [14, 15]. The commonly evaluated outcome is the prevalence of anal neoplasia in women with a history of genital neoplasia, [4, 14, 15] and with only a small number of anal HSILs reported. In two of the larger studies in which MZN assessment was performed in women with genital neoplasia [14, 15], anal HSIL was diagnosed in 6% (28/481) [14] and 8% (17/205) [15] of the cohorts respectively.

The vast majority of clinicians undertaking colposcopy and vulval examinations in the U.K. have not received training in high-resolution anoscopy (HRA), and it is not currently clear which patients might benefit from HRA assessments. Suggestions have been made for the need to ask about anal symptoms, and the benefit of digital anorectal examination (DARE) in patients with genital HSIL/cancer [16]. HRA has a long learning curve [17] and patients need to be aware of any tangible benefits before consenting to a full examination. However, the acceptability of HRA in patients attending a tertiary referral centre is high [18].

In this study, the high-resolution multizonal assessments were carried out by high-resolution anoscopy trained practitioners, including gynaecologists, colorectal surgeons and sexual health/ infectious disease specialists. All anogenital sites were considered, and only HSIL and/or cancer were evaluated for the outcome. In addition, information on follow-up, and data on new lesion development have been provided; similar data have not been available from previous studies. We describe a cohort that differs from those previously described in the literature; the study took place in a referral centre for the management of anogenital neoplasia in men and women, and as such 75% gave a prior history of HSIL or cancer in the anogenital region.

Full anogenital high-resolution evaluation allowed the identification of lesions that in many cases were unsuspected, in zones that had previously not been examined. At least one site of HSIL/cancer was diagnosed in 42% of the women at first visit, with 21% of these cases being a new site of disease (including six patients with anogenital cancer at a new site). The risk of developing a new lesion was also high; 17% of the women had a new HSIL/cancer diagnosed during follow-up (four patients with a new site of anogenital cancer), reinforcing the need for continued long-term surveillance. In women with MZN diagnosed at the first visit or during follow-up, the most common site of involvement was the perianus. This site has not been well described in previous studies reporting anogenital neoplasia in women. This study indicates the importance of considering the perianus separately in intraepithelial neoplasia and cancer risk in women.

Several factors were identified as posing a risk for developing MZN in our cohort. Ninety-eight percent of the women with MZN had a previous history of anogenital HSIL/cancer in one of the anogenital zones. The only woman diagnosed with MZN who did not have a prior history of HSIL/cancer was a current smoker. Several studies have already shown that current smoking is associated with an increased risk for anogenital cancers [19] and anal HSIL [20, 21]. Some studies found an association with the number of cigarettes smoked [19, 20], although this was not shown in our study. For most anogenital cancer sites, the association with previous smoking was substantially lower (or none) than for current smoking [19, 22]. HIV-positivity and pharmacological immunosuppression, especially in transplant recipients, are well-recognized risk factors for anogenital cancers. In a meta-analysis, the risk for vulval and vaginal cancers seemed to be substantially lower in HIV-positive patients in comparison to transplant recipients, unlike cervical and anal cancers [23].

There is a clear distinction in the referral patterns between patients with an index HSIL/cancer of the cervix when compared to other sites, with ‘cervical’ patients referred much later. This may be a reflection of the natural history of HPV-related anogenital neoplasia development. A large population-based cohort study in Sweden [5] showed that, after the first year of CIN3 diagnosis, the incidence rate ratios (IRR) for vaginal and vulval cancers fell over successive years, whereas an increasing IRR with time was observed for anal SCC. Similarly, other studies [10], found the increased risk for anal SCC was only significant ten or more years after the cervical cancer diagnosis. In women with cervical intraepithelial neoplasia (CIN3), the risk of developing anal cancer is higher than that of vulval cancer [7, 9]. Cervical cancer is the fourth most common cancer amongst women, whose incidence has been declining in developed countries, as a reflex of screening, and increasing in developing countries [24]. Vulval squamous cell carcinoma incidence has also been increasing, especially in younger women [25]. A recent meta-analysis [26] had shown that women with vulval cancer are one of the highest risk groups for anal cancer, with an incidence rate (IR) of 48 per 100 000 person years. For women with cervical cancer and vaginal cancer the anal cancer risk was lower than for the vulva, but also with a high incidence, with an IR of 9 per 100 000 persons year for the cervix and 10 per 100 000 persons year for the vagina.

A major strength of this study is the large number of cases with HSIL/cancer, facilitating analyses to identify groups at substantial risk of anogenital neoplasia. All women were evaluated using the same homogeneous protocol, in a service with experience in assessing these patients. A clear distinction between anal and perianal disease was made and reported accordingly. There was follow-up information available for the majority of patients (73%), enabling additional new diagnoses to be ascertained, unlike published cross-sectional studies showing data from a single time point.

There are, however, several limitations. This is a retrospective study, and there are some missing data on smoking status and CD4 nadir. The study was conducted in a tertiary referral centre, and thus the women that were referred tend to have a high disease burden, which may limit the generalizability of the results to other settings. There are no data on the anal HPV status of the patients nor that of their lesions, as HPV genotyping is not yet routinely performed in the UK anal neoplasia setting, as opposed to in colposcopy. For immunomodulators/immunosuppressive therapy, the heterogeneity of this population regarding their disorders and the multiplicity of drugs used restricted further sub-analysis. A small number of women had had their cervical examinations at a separate occasion to the rest of the MZN examination, leading to possible underestimation of cervical HSIL.

## Conclusions

Multizonal anogenital assessment facilitates diagnosis of occult areas of HSIL and cancer in high-risk women. Our data serve to possibly identify those women who may need this assessment, and that there is a subset of patients who are likely to be at particularly high risk, who will also benefit from further research. Multizonal assessment should be considered in the pharmacologically immunocompromised and those with a previous history of anogenital HSIL/cancer, especially current smokers. The occurrence of simultaneous HSIL/SCC in all anogenital zones is emphasised by this paper. Future training of clinicians, able to undertake a competent MZN evaluation, might be important to facilitate optimal patient management.

## Supplementary Information

**Additional file 1: Figure S1.** study flowchart.

## Data Availability

The anonymized dataset used and analysed during the current study is available from the corresponding author on reasonable request.

## Abbreviations

AIN: Anal intraepithelial neoplasia
CI: Confidence interval
CIN: Cervical intraepithelial neoplasia
HPV: Human papillomavirus
HRA: High-resolution anoscopy
HSIL: High-grade squamous intraepithelial lesions
IQR: Interquartile range
LAST: Lower Anogenital Squamous Terminology
LSIL: Low-grade anal squamous intraepithelial lesions
MZN: Multizonal anogenital neoplasia
RR: Relative risk
SD: Standard deviation
