# Supplementary material for: Multizonal anogenital neoplasia in women: a cohort analysis
Source: BMC Cancer. 2021 Mar 6;21:232. doi: 10.1186/s12885-021-07949-8 (PMC7937256; doi:10.1186/s12885-021-07949-8)
Supplement: Supplementary file 1 — Additional file 1: Figure S1. study flowchart. [file 12885_2021_7949_MOESM1_ESM.docx]

**Multizonal anogenital neoplasia in women: a cohort analysis**

Multizonal anogenital neoplasia

Andreia Albuquerque^1^, Michelle Godfrey^2*^, Carmelina Cappello^1*^, Francesca Pesola^3*^, Julie Bowring^1^, Tamzin Cuming^1^, Anke De Masi^1^, Adam N Rosenthal^1,4^, Peter Sasieni^3^, Mayura Nathan^1^

1. Homerton Anal Neoplasia Service (HANS), Homerton University Hospital, London, UK
2. Department of Obstetrics and Gynaecology, Queen’s Hospital, London, UK
3. King’s College London, London, UK
4. University College Hospital NHS Foundation Trust, London, UK

*Michelle Godfrey, Carmelina Cappello, Francesca Pesola contributed equally to this study.

308 new cases of women assessed from January 2012 to March 2017

271 cases with information regarding multizonal evaluation

37 cases with no information regarding multizonal evaluation

253 cases with complete data on multizonal disease assessment at first visit and/or follow-up

18 cases with incomplete multizonal disease assessment data at first visit and/or follow-up

**Figure S1: study flowchart**
